# Lysophosphatidic Acid Signaling in Diabetic Nephropathy

**DOI:** 10.3390/ijms20112850

**Published:** 2019-06-11

**Authors:** Jong Han Lee, Donghee Kim, Yoon Sin Oh, Hee-Sook Jun

**Affiliations:** 1 College of Pharmacy, Gachon University, Incheon 21936, Korea; jhleecw@gachon.ac.kr; 2 Lee Gil Ya Cancer and Diabetes Institute, Gachon University, Incheon 21999, Korea; dh2388@gachon.ac.kr; 3 Department of Food and Nutrition, Eulji University, Seongnam 13135, Korea; ysoh@eulji.ac.kr; 4 Gachon University Gil Medical Center, Gachon Medical and Convergence Institute, Incheon 21565, Korea

**Keywords:** diabetic nephropathy, lysophosphatidic acid, lysophosphatidic acid receptor, chronic kidney injury

## Abstract

Lysophosphatidic acid (LPA) is a bioactive phospholipid present in most tissues and body fluids. LPA acts through specific LPA receptors (LPAR1 to LPAR6) coupled with G protein. LPA binds to receptors and activates multiple cellular signaling pathways, subsequently exerting various biological functions, such as cell proliferation, migration, and apoptosis. LPA also induces cell damage through complex overlapping pathways, including the generation of reactive oxygen species, inflammatory cytokines, and fibrosis. Several reports indicate that the LPA–LPAR axis plays an important role in various diseases, including kidney disease, lung fibrosis, and cancer. Diabetic nephropathy (DN) is one of the most common diabetic complications and the main risk factor for chronic kidney diseases, which mostly progress to end-stage renal disease. There is also growing evidence indicating that the LPA–LPAR axis also plays an important role in inducing pathological alterations of cell structure and function in the kidneys. In this review, we will discuss key mediators or signaling pathways activated by LPA and summarize recent research findings associated with DN.

## 1. Introduction

Diabetic nephropathy (DN) is a microvascular complication of diabetes and develops in approximately 20–40% patients with diabetes, including type 1 and type 2 diabetes patients [1,2]. It has become the main risk factor for the development of chronic kidney diseases, such that most DN patients progress to end-stage renal disease (ESRD) and require renal replacement in the end [2]. Thus, DN not only increases the health cost for individuals and the society at large but is also a risk factor for morbidity and mortality. Intensive glycemic control is a well-established strategy for the prevention of DN. Despite the efforts for the management of hyperglycemia and hypertension using current therapies, such as angiotensin converting enzyme (ACE) inhibitors and angiotensin II receptor blockers (ARBs) [3,4], the risk of DN progression has still not been reduced. Currently available drugs only delay the progress of the disease, rather than curing it. Therefore, a novel and effective therapeutic approach is urgently needed for patients with DN.

Phospholipids are generally known to be structural components of plasma membranes, but growing evidence indicates that membrane phospholipids also play crucial roles as signaling molecules and exert a wide range of physiological responses. Lysophosphatidic acid (LPA) is a small, naturally occurring glycerophospholipid, which is composed of a glycerol backbone with an ester-linked acyl chain and a phosphate group. It is also produced by the action of various lysophospholipases, including autotaxin (ATX), and phospholipases A1 or A2 (PLA1 and PLA2). LPA acts through specific LPA receptors (LPAR1 to LPAR6) coupled with G protein and is associated with a wide range of cell responses, such as proliferation and migration [5,6], and pathologies of a number of diseases, including fibrosis, cancer, neuronal disorders, and bone metabolism [5,7,8,9,10]. Hyperglycemia, advanced glycation end product (AGE), and pro-inflammatory cytokines are considered the three main initial mediators of DN development [11,12]. However, some recent studies also showed that the LPA–LPA receptor axis may play an important role in the pathogenesis of diabetic kidney disease.

In this review article, we review the key mediators and signaling pathways activated by LPA and summarize newly reported signaling pathways modulated by different LPA receptor antagonists in different DN models.

## 2. Biosynthesis and Degradation of LPA

**Figure 1:**
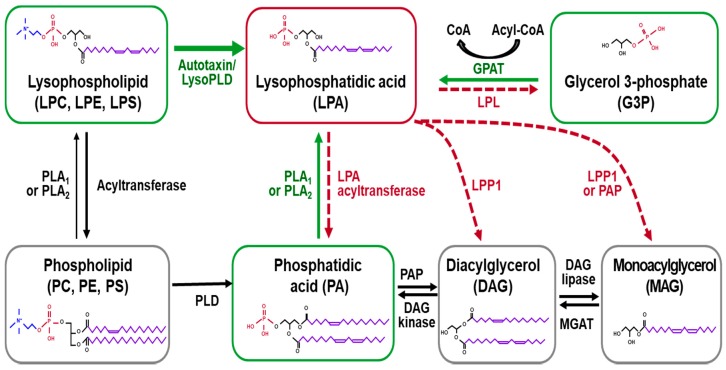
The enzymatic pathways of lysophosphatidic acid (LPA) synthesis and degradation. LPA can be synthesized from different precursors, including lysophospholipids, phosphatidic acid, and glycerol 3-phosphate. The enzymes and pathways involved in LPA production are indicated using green text and a green solid line, respectively. LPA is converted into either monoacylglycerol or phosphatidic acid. The enzymes and pathways involved in LPA conversion are indicated using red text and a red dotted line, respectively. Lysophosphatidylcholine (LPC), lysophosphatidylethanolamine (LPE), lysophosphatidylserine (LPS), lysophospholipase D (Lyso PLD), lysophospholipase (LPL), glycerol 3-phosphate acyltransferase (GPAT), phospholipase C (PLC), phospholipase A1 or A2 (PLA1 and PLA2), diacylglycerol (DAG), monoacylglycerol (MAG), MAG acyltransferase (MGAT), lipid phosphate phosphatase 1 or 2 (LPP1 and LPP2), phosphatidate phosphatase (PAP), phosphatidylcholine (PC), phosphatidylethanolamine (PE), phosphatidylserine (PS), and phospholipase D (PLD).

LPA is the smallest bioactive lysophospholipid (MW 430–480 Da) derived from membrane phospholipids [13] and acts as an extracellular signaling molecule via its receptors, regulating various cellular processes, including cell proliferation, survival, migration, differentiation, remodeling, and cytokine/chemokine secretion [13]. LPA is known to be water soluble and is present in most tissues and biological fluids, such as plasma, saliva, tears, follicular fluid, and cerebrospinal fluid [5,14]. LPA is produced by several enzymes, either in the intracellular or extracellular compartment, as shown in Figure 1. In the intracellular compartment, LPA is naturally synthesized through the action of glycerol-3-phosphate acyltransferase (GPAT) during the process of triglyceride and phospholipid anabolism, which is found mainly in the mitochondria and endoplasmic reticulum of various cell types [15,16]. LPA is also synthesized by the action of intra/extra-cellular PLA1 or PLA2 from phosphatidic acid (PA). Ubiquitously expressed PLA1 and PLA2 in the body hydrolyze the bond between the fatty acid chain and the glycerol backbone at *sn1* (saturated) or *sn2* (unsaturated) positions of PA [17,18]. Membrane phospholipids are major sources for LPA production. Metabolically, they are first converted into lysophospholipids, such as lysophosphatidylethanolamine (LPE), lysophosphatidylcholine (LPC), lysophosphatidylserine (LPS), and then, subsequently cleaved by ATX, also known as ectonucleotidepyrophosphatases/ phosphodiesterases-2. This pathway is considered a determinant for the LPA level in plasma [19,20].

LPA is converted by several classes of enzymes, including lipid phosphate phosphatases (LPPs), LPA acyltransferase, and phospholipases [21,22,23], as shown in Figure 1. LPPs (LPP1, LPP2, and LPP3) exist extracellularly and intracellularly in the endoplasmic reticulum and Golgi, and dephosphorylate LPA and degrade it into monoacylglycerol (MAG). LPA can also be converted to PA by the action of the acylglycerophosphate acyltransferase (AGPAT) enzyme, also known as LPA acyltransferase [24]. The third alternative pathway for LPA degradation is mediated by the action of lysophospholipases, via formation of glycerol-3-phosphate [25].

## 3. LPA Receptors and Intracellular Signaling Pathways

**Figure 2:**
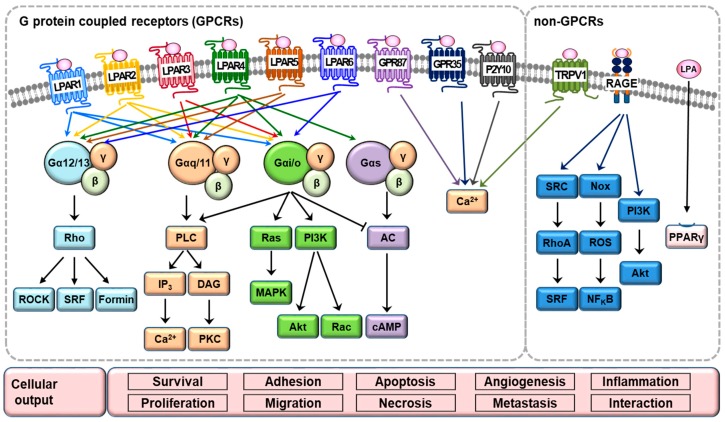
LPA signaling pathways. LPA can induce multiple cellular effects via binding to specific GPCRs, including LPAR1–LPAR6, as well as binding to non-GPCRs, such as transient receptor potential vanilloid 1 (TRPV1), receptor for advanced glycation end products (RAGE), and intracellular peroxisome proliferator-activated receptor gamma (PPARγ). After binding to receptors, LPA activates downstream intracellular signaling pathways, thereby resulting in various physiological and pathophysiological responses, as described in detail in the text.

LPA induces various cellular effects by binding to specific G protein-coupled LPA receptors (LPARs) and activates downstream intracellular signaling pathways, resulting in various physiological and pathophysiological responses [5]. Till now, six LPARs have been identified and classified into A rhodopsin-like G protein-coupled receptors [26]. They can be further grouped into two groups, according to their distinct protein homology, such as LPAR1 to LPAR3 belonging to the endothelial differentiation gene (*Edg*) family, and LPAR4 to LPAR6 belonging to the *P2Y* purinergic gene cluster [6,8]. These receptors have the ability to interact with at least one or more heterotrimeric G subunits, such as Gαi/o, Gαq/11, Gα12/13, and Gs [6]. LPAR1/Edg2 and LPAR2/Edg4 receptors couple with Gαi/o, Gαq/11, and Gα12/13. Once bound together, the complexes transduce extracellular signals into intracellular pathways through molecules, such as the Ras homologous (Rho) protein family of GTPases, phospholipase C (PLC), diacylglycerol (DAG), mitogen-activated protein kinase (MAPK), and phosphatidylinositol 3-kinase (PI3K)-protein kinase B (Akt). Activation of these receptors mostly ends up promoting cell proliferation, survival, and migration [27,28]. LPAR3/Edg7 couples with Gαi/o and Gαq/11, and participates in LPA-induced Ca^2+^ mobilization, PLC, adenylyl-cyclase inhibition, and MAPK activation [28]. LPAR4/GPR23/P2Y9 and LPAR5/GPR92 induce stress fiber formation and neurite retraction through Gα12/13 and downstream Rho/Rho-associated protein kinase (ROCK) pathway [29,30]. LPAR4 is known as the only LPA receptor that can increase intracellular cAMP accumulation by coupling to Gs [29]. LPAR5 interacts with Gαq/11 and increases intracellular Ca^2+^ levels [31]. LPA6/P2Y5 receptor binds to either Gαi/o or Gα12/13, and induces Rho-dependent alteration of cellular morphology [32,33]. In addition, other G protein-coupled receptors, including GPR35 [34] and P2Y10 [35], were also identified as LPARs, which induced Ca^2+^ responses by LPA stimulation. LPA can also bind to and activate non-GPCR targets, the receptor for advanced glycation end products (RAGE) [36], and the cation channel transient receptor potential vanilloid 1 (TRPV1) [37]. RAGE participates in LPA-induced nicotinamide adenine dinucleotide phosphate (NADPH) oxidase (Nox), reactive oxygen species (ROS) induction, and activation of NF_K_B, serum response factor (SRF), PI3K, and Akt [38]. TRPV1 increases intracellular Ca^2+^ levels following LPA stimulation [37]. Another non-GPCR, peroxisome proliferator-activated receptor gamma (PPARγ), is the intracellular receptor for LPA, and is critically important for mediating the effects of LPA on vascular remodeling [39]. Among them, the expression of LPAR1-4 has been detected in renal tissue [40]. Although many LPA receptors and their signaling pathways have been identified, as shown in Figure 2, the functional role of each receptor is poorly understood. Further studies will be required.

## 4. Pathogenesis of DN

### 4.1. Definition of Chronic Kidney Disease

Kidney disease is a heterogeneous group of disorders affecting kidney structure and function, which can be further classified into two distinct syndromes, acute and chronic kidney disease (CKD) [6]. Acute kidney injury is characterized by rapid diminution of kidney function, resulting in excretion of creatinine [41]. It occurs over several hours or days but not more than three months, whereas chronic kidney injury is in cases of more than three months. Acute kidney injury often leads to the development of chronic kidney failure [41]. Conversely, chronic kidney disease increases the risk of incidence of acute kidney injury [42,43]. Whatever the initial injury, the final pathogenesis of CKD involves renal fibrosis, characterized by excessive extracellular matrix (ECM) accumulation in glomerular and tubular interstitial cells [44,45]. Renal fibrosis changes tissue architecture and function, which leads to kidney dysfunction and failure.

### 4.2. Glomerulosclerosis

Glomerulosclerosis is hardening of glomeruli in the kidney and is frequently referred to as focal segmental glomerulosclerosis or nodular glomerulosclerosis [46]. The initial pathogenic events start in glomerular endothelium, including the activation and dysfunction of endothelial cells, hemodynamic alterations, as well as loss of glomerular basement membrane (GBM) electrical charge. These alterations subsequently trigger an inflammatory response in the glomerular apparatus, which further affects mesangial cells and induces proliferation and dysfunction of mesangial cells [43,47]. Excessive ECM production is the underlying mechanism for mesangial expansion. Podocytes are the cells located inside the Bowman’s capsule of the kidney, which anatomically wrap around capillaries of the glomerulus [48]. The damage in podocytes decreases their number and increases foot process effacement and denudation of GBM. The accumulated damage increases cell death by apoptosis or necrosis, and replacement of glomerular cells by ECM, which, in turn, increases hardening of glomeruli and eventually leads to glomerulosclerosis [16,44].

### 4.3. Tubular Interstitial Fibrosis

Although the initial glomerular damage and glomerulosclerosis are the major factors for the development of CKDs, the fibrosis in the tubulo-interstitial compartment is also a major contributor [43,49]. Initial glomerular damage increases the leakage of abnormally filtered proteins, including albumin, complement, and cytokines. These proteins and immune effectors or molecules activate tubular cells and initiate an inflammatory response. Simultaneously, different types of inflammatory cells (macrophages, monocytes, lymphocytes, mast cells, and dendritic cells) are recruited from circulation by increasing the expression of chemokines (monocyte chemoattractant protein 1, CCL2/MCP1; regulated upon activation, normal T cell expressed, and secreted, CCL5/RANTES), and adhesion molecules (E and P selectins, intercellular adhesion molecule-1, ICAM-1; vascular cell adhesion molecule-1, VCAM-1) [50]. In particular, the recruited monocytes transdifferentiate into macrophages in the kidney interstitium. Myofibroblasts are responsible for producing fibrillar matrix in the renal interstitium [51]. Under normal conditions, a lower population of myofibroblasts is maintained. However, they are activated, and their number is increased through various ways (proliferation, epithelial mesenchymal transition (EMT), and recruitment of circulating fibrocytes derived from bone marrow cells) in a pathological environment. In addition, accumulation of ECM proteins within the kidney interstitium is the consequence of imbalance between synthesis and degradation of ECM. Thus, the expression of plasminogen activator inhibitor-1 (PAI-1, a specific inhibitor of urokinase-type and tissue-type plasminogen activator (uPA and tPA)) and tissue inhibitor of metalloproteinase 1 (tissue inhibitor matrix metalloproteinase-1, TIMP-1; a specific inhibitor of MMPs) are increased during fibrosis, resulting in the inhibition of ECM degradation [52,53,54]. Beyond the abovementioned pathological events, hemodynamic alterations also reduce post-glomerular blood flow to peritubular capillaries, which, in turn, leads to hypoxic conditions and damage to tubular epithelium [55].

## 5. Cellular Signaling Pathways Involved in Pathogenesis of DN

In the diabetic milieu, such as hyperglycemia and dyslipidemia, metabolic alteration increases the production of ROS mainly through the Polyol and/or Nox pathway [56,57]. In addition, an increased intracellular glucose level increases AGE production and ROS by activating the AGEs receptor (RAGE) axis [58]. These abnormal metabolic and physiological consequences directly induce both vascular endothelial cell dysfunction and hemodynamic alterations, including activation of the renin–angiotensin system. Subsequently, this impact triggers a number of cellular signaling cascades, including the protein kinase c (PKC), mitogen-activated protein kinases (MAPKs; p38 and c-Jun N-terminal kinases (JNK)), Janus kinase/signal transducer and activator of transcription protein (JAK/STAT), and transforming growth factor (TGF)-β/Smad, thereby inducing a cellular response via activation of key transcription factors, such as NF-κB [56,57]. In particular, several vasoactive factors (angiotensin II, thromboxane, and endothelin-1) enhance their fibrotic action in diabetic renal diseases via secondary induction of TGF-β expression [56,59].

The Smad signaling pathway has also been reported to be associated with the renal hypertrophy and accumulation of ECM molecules through TGF-β signaling in DN [56,60,61]. Accumulation of AGEs within the glomerular apparatus, mesangial matrix, and tubular cells also directly causes serious alteration of diabetic kidney structure by reacting with plasma proteins and extra vascular proteins. As a consequence, AGEs lead to the transcriptional upregulation of TGF-β1, IL-6, and NF-κB, possibly via activation of PKC and/or oxidative stress [59,62]. PKCs are important players for the onset and progression of DN via hyperglycemia-induced upregulation of vascular endothelial growth factor (VEGF) expression in the mesangial cells [63]. In response to such molecules or signals, chemokines, growth factors, and profibrotic factors are ultimately upregulated in renal cells, such as tubular epithelial cells, podocytes, and mesangial cells, which contribute to cellular injury, progressive fibrosis, and loss of glomerular filtration rate, thereby increasing proteinuria during the development and progression of DN [56,57].

Recently, new molecules or potential signaling pathways have been added to the established pathways, which has made the pathogenesis of DN more complex. However, new observations simultaneously increase the possibility of identifying new targets for the treatment of DN. Interleukin (IL)-33-mediated suppression of the tumorigenicity 2 receptor (ST2) axis is one such pathway, which is very attractive due to its association with inflammatory response. IL-33 was first identified as a member of the IL-1 family in 2005 [64]. IL-33 acts through ST2 [65], which plays an important role in the immunity against pathogens, type 2 inflammation, tissue homeostasis, and repair [66,67]. Soluble ST2 level elevates in the serum of CKD and is associated with the severity of the disease [68], possibly suggesting its role in the development of CKD. However, these studies are very limited.

Sodium–glucose cotransporter (SGLT)-2 may be a potential contributor to CKD. Under normal physiological conditions, urine does not contain glucose, owing to its effective reabsorption by two transporters, SGLT-1 and SGLT-2 [69]. The SGLT-2 transporter is located on the luminal side of the first segment of the proximal tubule in the kidney. It is a high-capacity, low-affinity transporter, but is responsible for the reabsorption of approximately 90% of all filtered glucose [70]. SGLT-2 inhibitor induces glycosuria and natriuresis by blocking reabsorption of glucose and sodium in the proximal tubule [71]. As a consequence, afferent arteriolar vasoconstriction is induced, thereby reducing intraglomerular pressure, decreasing hyperfiltration, and improving renal function [72].

Current evidence also suggests that autophagy is critical in kidney physiology and homeostasis. In the diabetic milieu, increased oxidative stress, inflammation, and mitochondrial dysfunction modulate the autophagy activation and inhibition as well as lead to cellular recycling dysfunction [73]. Reduction of autophagy induces loss of podocytes, damage in proximal tubular cells, and glomerulosclerosis [74,75]. High glucose treatment has activated autophagy in podocytes and protected the podocytes from hyperglycemia-related apoptosis [76]. Similarly, autophagy related 5 (Atg5)-knockout diabetic mice (deficiency of autophagy activation) showed more severe proteinuria and impaired renal function [77,78]. Autophagy also protects mesangial cells from apoptosis induced by TGF-β1 via transforming growth factor β-activated kinase (TAK)1 and PI3K–AKT-dependent pathways [79]. Several studies have consistently indicated that DN is associated with decreased autophagy and increased apoptosis [80,81,82].

## 6. Chronic Kidney Injury and LPA–LPAR Axis

CKD has been becoming a major public health problem globally. It involves a progressive loss of kidney function over a period of months or years, which frequently leads to end-stage renal failure [1]. The main risk factors of CKD include high blood pressure, diabetes, cardiac disease, and a family history of kidney failure with genetic problems [83]. The potential role of LPA in the pathogenesis of kidney disease was suggested for the first time following the discovery of a positive correlation between circulation LPA levels and renal dysfunction in patients with kidney disorder at the end of the 1990s [84]. Later on, Grove KJ et al. also reported that the LPA level was elevated in glomeruli of eNOS (–/–) *db/db* mice, a robust model of DN [85]. Furthermore, LPA induced renal tubulointerstitial fibrosis, a classical hallmark of CKD, in a mouse model of unilateral ureteral obstruction [86]. More recently, LPA was identified as a biomarker of CKD in metabolic screening assay, using plasma samples from CKD patients with diverse etiologies and two different CKD rodent models [87]. Consistent with results of other studies, the LPA and LPC forms (16:0, 18:0, 18:1, and 18:2) were also detected in urine of DN patients [88]. On the contrary, several studies showed similar or reduced LPA levels in the plasma, but an increased urinary LPA level in CKD patients and animal models, suggesting that the local expression levels of LPA and LPARs are more important for the development of CKD [88,89,90]. LPA regulates various biological responses by binding to G-protein–coupled receptors (LPAR1–LPAR6) [5].

Recently, we and another group reported that the LPAR1 and/or LPAR3 were upregulated in different DN mouse models [91,92,93]. Treatment with a dual LPAR1/3 antagonist (Ki16425 or BMS002) or AM095 (a novel antagonist of LPAR1) reduced renal injury in the *db/db* mice (leptin receptor-deficient mouse with human type 2 diabetic phenotype) and streptozotocin (STZ)-induced diabetic mice (pancreatic beta cell-destroyed mouse with human type 1 diabetic phenotype) [91,92,93]. The expression of ATX and LPA production increases and activates the LPAR1-mediated signaling pathway in mesangial cells’ exposure to high-glucose media and in the kidney cortex of diabetic *db/db* mice [91]. LPA–LPAR1 activation increases the phosphorylation of glycogen synthase kinase (GSK)3β at serine 9 residue (Ser9) and induces translocation of sterol regulatory element-binding protein (SREBP) 1 into the nucleus [91]. Subsequently, it induces TGF-β expression, which contributes to the development of glomerular injury in *db/db* mice. However, treatment of ki16425 reduces proteinuria, glomerular tuft area and volume, and mesangial matrix expansion by regulating the LPA–GSK3β–SREBP1 axis [91]. In line with our observation, LPAR1 deletion in unilateral ureteral obstruction-induced mice prevents renal fibrosis by suppressing the expression of connective tissue growth factor in proximal tubular epithelial cells [86]. Additionally, Diao et al. reported that LPAR3 deletion affects the spatiotemporal expression of collagen types I, III, IV, and VI in peri-implantation of the mouse uterus [94]. All of these studies suggest that the LPA–LPAR axis regulates renal fibrosis differently for different kidney cell types or different tissues, and that at least two receptors, LPAR1 and LPAR3, might be important contributors to renal fibrosis.

Similarly, Zhang et al. showed improvements in renal function, using a different dual LPAR1/3 antagonist, BMS002, in the endothelial nitric oxide synthase-deficient (eNOS (–/–)) *db/db* mouse [92]. The expressions of ATX, LPAR1, and LPAR3 proteins in these mice significantly increased in the glomerular podocytes and tubular epithelial cells in the renal cortex [92]. Blockading the LPAR1/3 activity ameliorated glomerular injury and reverses kidney dysfunction by reducing podocyte loss and increased the phosphorylation of Akt2 (known to be essential for maintaining podocyte viability and function) [95], which prevented reduction in the glomerular filtration rate and reduced proteinuria without affecting blood pressure [92]. However, the underlying molecular mechanism in their study was not fully addressed and further studies will be needed.

Furthermore, our studies showed that increased LPA levels in STZ-induced diabetic mice increases Toll-like receptor (TLR) 4 expression and directly activates the NF-κB, the master transcription factor responsible for inflammatory cytokine expression. Simultaneously, activated TLR4 also produces ROS through the nicotinamide adenine dinucleotide phosphate (NADPH) oxidase system [93]. All these intracellular changes synergistically activate NF-κB and/or JNK by increasing phosphorylation of p65 and JNK [93]. Ultimately, it leads to renal fibrosis via upregulation of pro-inflammatory cytokines and fibrotic factors, including TGF-β1, TIMP-1, and fibronectin. In contrast, AM095 treatment attenuates DN by downregulation of these signaling pathways in vivo. In mesangial cells, LPA treatment activates these signaling pathways similar to that in mice; however, this degree of activation was reduced by AM095 treatment [93]. We also observed some beneficial effects of AM095 treatment on glycemic control. However, the underlying mechanism should be addressed in future studies.

Mesangial cell proliferation and accumulation in the pathogenesis of DN is a major risk factor contributing to glomerulosclerosis. LPA treatment increased the proliferation of mouse mesangial cells (SV40 MES13), concomitant with the increased expression levels of cyclin D1 and CDK4 and decreased expression of p27^Kip1^. The expression of Krüppel-like factor 5 (KLF5) was upregulated by activating MAPK and elevating the expression of early growth response 1 (Egr1) in the kidney cortex of *db/db* mice and LPA-treated SV40 MES13 cells. Moreover, LPA significantly increased the activity of Rac1 GTPase in SV40 MES13 cells, and the dominant–negative form of Rac1 blunted the phosphorylation of p38, the upregulation of Egr1, and the LPA-mediated induction of KLF5, indicating that the downstream pathway of Rac1 was involved in LPA-induced mesangial cell proliferation [96]. Taken together, these observations suggested that the Rac1/MAPK/KLF5 signaling pathway is one of the underlying mechanisms contributing to glomerular hyper proliferation during the progression of DN. Recently, Guo et al. showed that LPA activates β-catenin, a downstream mediator of Wnt signaling, in colon cancer cells and KLF5 plays a critical role in β-catenin activation [97]. Several studies directly indicated that the Wnt/β-catenin signaling pathway was implicated in renal fibrosis and apoptosis in CKD models [98,99]. Thus, the regulation of these pathways may provide a potential therapeutic target for the treatment of DN.

Some studies have also suggested that LPA may bind to the receptor for RAGE, a member of the immunoglobulin superfamily [36,100]. Under in vitro conditions, this interaction is essential for LPA-induced ovarian tumor implantation and metastasis, as well as for LPA-mediated proliferation and migration of vascular smooth muscle cells [36]. In addition, LPA failed to activate vascular Akt signaling in mice that were administered soluble RAGE or genetic deletion of RAGE [36]. These observations indicated that RAGE-mediated signal transduction may play an important role in diabetic microvascular complication, including DN. Zaslaysky et al. also revealed that three major LPARs (LPAR1-3) form homo- or hetero-dimers within the LPAR subgroup and hetero-dimers with sphingosine 1 phosphate receptor (S1PR), pH-sensing G protein-coupled receptor (GPR4), and ovarian cancer G-protein coupled receptor-1 (OGR1/GPR68) [101], thereby activating the downstream signaling pathways linked to inflammation and fibrosis. S1PR signaling activation promotes renal fibrosis in the diabetic condition [102,103,104]. In the terminal ileum of inflammatory bowel diseases, the expression of OGR1/GPR68 positively increases the expression of pro-fibrotic genes and collagen deposition, suggesting the potential involvement of these receptors, signaling for the pathogenesis of DN [105]. However, there is no direct evidence connecting DN pathogenesis with dimer formation between LPARs and these receptors. Another potential LPA receptor is the TRPV1 ion channel [37]. However, there have been no reports demonstrating its role in DN.

## 7. Conclusions and Future Research Directions

**Figure 3:**
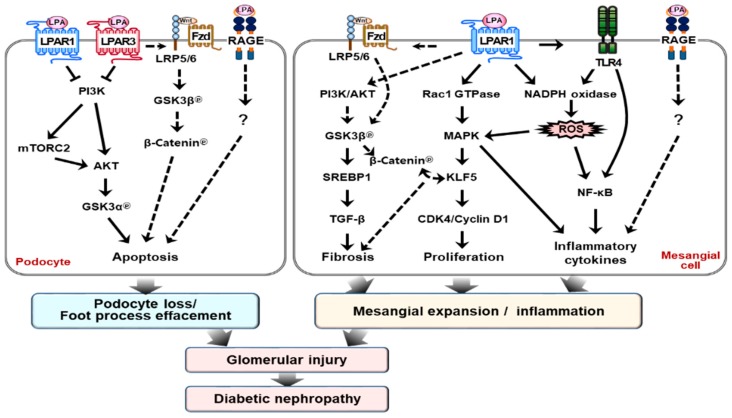
Schematic representation of LPA signaling in diabetic nephropathy models.

DN is the main risk factor for chronic kidney diseases, which mostly progress to the development of ESRD in the end. The best treatment regimen for DN is kidney replacement; however, it is highly restricted, due to various reasons, such as rare donors and rejection of the transplanted organ. Several medications are currently available for DN, but they are not sufficient for the recovery from kidney injury and restoration of kidney function in DN patients. Alternative drugs are thus required. Accumulated evidence indicates that the LPA–LPAR axis plays an important role in inducing pathological alterations of cell structure and function in the kidneys. Current studies showed that LPAR antagonism using pharmacological inhibitors significantly decreases the abnormality of kidney structure, such as GBM thickness, and increases of renal function, such as reducing proteinuria in different types of diabetic mouse models by regulating several signaling pathways, as shown in Figure 3.

The currently available data suggest that LPA signaling may regulate fibrosis, proliferation, and the inflammatory response in mesangial cells and podocytes or induce apoptosis via the following signaling pathways: 1) PI3K-AKT-GSK3β-TGF-β axis for fibrosis; 2) Rac1GTPase-MAPK-KLF5-CDK4/Cyclin D1 axis for proliferation; 3) TLR4-NADPH oxidase-ROS–NF-_K_B/MAPK or TLR4-NF-_K_B/MAPK axis for inflammatory response; 4) PI3K-AKT-GSK3α axis for apoptosis; 5) LPA-RAGE axis for glomerular injury; and 6) Wnt/β-catenin axis for fibrosis and apoptosis. For further details, please see above. LPAR: LPA receptor, RAGE: AGE receptor, Rac1: Ras-related C3 botulinum toxin substrate 1, NADPH: Nicotinamide adenine dinucleotide phosphate oxidase, TLR4: Toll-like receptor 4, NF-_K_B: Nuclear factor-_K_B, KLF5: Krüppel-like factor 5.

However, there are still many unanswered questions. Further studies should be performed in the following directions: Since the kidney consists of heterogeneous cells (such as podocytes, mesangial microvascular endothelial cells, and renal proximal tubule epithelial cells) and the LPAR expression level may be different in different cell types, further studies should first determine the LPAR expression level under different physiological and pathophysiological conditions. In addition, the studies should investigate the effects of LPAR antagonism on each type of kidney cell, using not only pharmacological inhibitors, but also genetically engineered in vivo models. Moreover, there are many different forms of LPA, which exhibit different affinities for each LPAR type. Each LPAR also exhibits various interaction affinities with one or more G protein subunits, thereby promoting different biological effects. These aspects should be further elucidated. Furthermore, the LPA–LPAR-mediated signaling pathway is also modulated by a cross-talk with other signaling pathways, such as epidermal growth factor (EGF) or vasoactive (angiotensin II) receptor-mediated signals [106], which will be defined in future studies. Since LPA regulates local blood flow, systemic blood pressure, and platelet function, and these factors directly play a critical role in renal function [40,107], future studies need to investigate the effect of LPA on the physiological and pathophysiological conditions in the renal vasculature and the tubular system. In addition, there are no studies demonstrating the relationship between the LPA–LPAR axis and the IL-33-ST2 axis and SGLT2 and autophagy. These new avenues should also be investigated in the future. Further understanding of the precise mechanisms underlying LPA action under physiological and pathophysiological conditions may facilitate the development of new therapeutic targets for DN.

## Abbreviations

DN	Diabetic nephropathy
ESRD	End-stage renal disease
ACE	Angiotensin converting enzyme
ARBs	Angiotensin II receptor blockers
LPA	Lysophosphatidic acid
ATX	Autotaxin
AGE	Advanced glycation end (product)
GPAT	Glycerol-3-phosphate acyltransferase
PLA1 and PLA2	Phospholipases A1 or A2
PA	Phosphatidic acid
LPE	Lysophosphatidylethanolamine
LPC	Lysophosphatidylcholine
LPS	Lysophosphatidylserine
LPPs	Lipid phosphate phosphatases
MAG	Monoacylglycerol
AGPAT	Acylglycerophosphate acyltransferase
PLC	Phospholipase C
DAG	Diacylglycerol
MAPK	Mitogen-activated protein kinase
PI3K	Phosphatidylinositol 3 kinase
PKB	Protein kinase B
ROCK	Rho-associated protein kinase
RAGE	Receptor for advanced glycation end products
Nox	NADPH oxidase
PPARγ	Peroxisome proliferator-activated receptor γ
CKD	Chronic kidney disease
ECM	Extracellular matrix
GBM	Glomerular basement membrane
EMT	Epithelial mesenchymal transition
CCL2/MCP1	Monocyte chemoattractant protein 1
CCL5/RANTES	Regulated upon activation, normal T cell expressed, and secreted
ICAM-1	Intercellular adhesion molecule-1
VCAM-1	Vascular cell adhesion molecule-1
PAI-1	Plasminogen activator inhibitor-1
uPA and tPA	Urokinase-type and tissue-type plasminogen activator
TIMP-1	Tissue inhibitor matrix metalloproteinase-1
JNK	c-Jun N-terminal kinases
VEGF	Vascular endothelial growth factor
SGLT-2	Sodium–glucose cotransporter-2
GSK3β	Glycogen synthase kinase 3β
SREBP 1	Sterol regulatory element-binding protein1
TGF-β	Transforming growth factor-β
TLR 4	Toll-like receptor 4
NF-_K_B	Nuclear factor-_K_B
KLF5	Krüppel-like factor 5
Egr1	Early growth response 1
S1PR	Sphingosine 1 phosphate receptor
GPR4	pH-sensing G protein-coupled receptor
OGR1/GPR68	Ovarian cancer G-protein coupled receptor-1
